# HDAC4 is expressed on multiple T cell lineages but dispensable for their development and function

**DOI:** 10.18632/oncotarget.15077

**Published:** 2017-02-03

**Authors:** Queping Liu, Xilin Zhang, Congcong Yin, Xiang Chen, Zhenggang Zhang, Stephen Brown, Hongfu Xie, Li Zhou, Qing-Sheng Mi

**Affiliations:** ^1^ Department of Dermatology, Xiang-Ya Hospital of Central South University, Changsha, Hunan, China; ^2^ Henry Ford Immunology Program, Henry Ford Health System, Detroit, MI, USA; ^3^ Department of Dermatology, Henry Ford Health System, Detroit, MI, USA; ^4^ Department of Neurology, Henry Ford Health System, Detroit, MI, USA; ^5^ Department of Radiation Oncology, Henry Ford Health System, Detroit, MI, USA; ^6^ Department of Internal Medicine, Henry Ford Health System, Detroit, MI, USA

**Keywords:** HDAC4, conventional T cells, invariant NKT cells, development, polarization, Immunology and Microbiology Section, Immune response, Immunity

## Abstract

Histone deacetylation, reciprocally mediated by histone deacetylases (HDAC) and acetyltransferases, represents one major form of post-translational modification. Previous research indicates that HDACs play an essential regulatory role in the development of various immune cells. However, the specific function of individual HDACs remains largely unexplored. HDAC4, a member of class II HDACs, profoundly investigated in the nervous system, while the expression profile and function of HDAC4 in T cells are barely known. For the first time, we report here that HDAC4 is expressed in the multiple T cell lineages. Using T-cell-specific HDAC4-deficient mice, we discovered that lack of HDAC4 did not alter the frequencies of conventional T cells, invariant NKT (iNKT) cells or regulatory T cells within both the thymus and secondary lymphoid organs. Moreover, conventional T cells and iNKT cells from wild-type and HDAC4-deficient mice displayed no significant difference in cytokine production. In conclusion, our results imply that under steady stage, HDAC4 is not required for the development and function of multiple T cell lineages, including conventional T cells and iNKT cells.

## INTRODUCTION

Originating from hematopoietic stem cells (HSC) in the bone marrow (BM), T lymphocytes develop in the thymus and become fully functional within the peripheral lymphoid tissues. Particularly, the rearrangement of T cell receptor (TCR) α-, β-, γ- and δ- chains drives double-negative T cells to turn into double-positive (DP) αβ T or γδ T cells. The DP αβ T lymphocytes which are further subjected to positive and negative selection, eventually develop into conventional single-positive (SP) CD4^+^ or CD8^+^ T cells. The CD4^+^CD25^+^Foxp3^+^ regulatory T (Treg) cells, specified by the transcription factor forkhead box P3 (Foxp3), which mainly regulate peripheral T cell tolerance, are critically involved in the pathogenesis of autoimmune diseases [1, 2]. Invariant natural killer T (iNKT) cells represent a subset of self-reactive T cells expressing Vα14-Jα18 (mouse) or Vα24-Jα18 (human) TCR chains that recognize lipid antigens presented by the MHC I-like CD1d molecule [3–6]. iNKT cells derive from conventional T cell lineage at the DP stage, and are selected by DP thymocytes expressing CD1d-loaded lipid antigens [7, 8]. After positive selection, iNKT cells proliferate and proceed through four distinct maturation stages phenotypically distinguished by CD44 and NK1.1 expressions: stage 0 iNKT cells are CD24^+^ precursors; stage 1 iNKT cells are CD44^-^NK1.1^-^ immature precursors; stage 2 iNKT cells are CD44^+^NK1.1^-^ semi-mature cells that primarily produce interleukin (IL)-4; and stage 3 iNKT cells are CD44^+^NK1.1^+^ mature iNKT cells that mainly secret interferon (IFN)-γ. Once activated, iNKT cells rapidly produce a large amount of cytokines, which enable their potent regulatory function in diverse immune responses, and therefore contribute to the pathogenesis of infectious, allergic and autoimmune diseases, and cancer [9, 10].

The proper generation of self-tolerant effector T cells are under the stringent control of both transcriptional and post-translational modifications. Research in the transcriptional gene regulation of thymic T cell development has resulted in the identification of several lineage-specific transcription factors, such as ThPOK [11], Runx3 [12] and PLZF [13, 14]. Also, post-translational gene modification, especially histone deacetylation, plays an essential role in T cell development and function [15–18]. Histone deacetylases (HDACs) are enzymes that regulate gene expression by removing acetyl residues from target histones or non-histone proteins and consequently modifying chromatin structure. HDACs are divided into four classes (class I: HDAC1, 2, 3 and 8; class II: HDAC4, 5, 6, 7, 9 and 10; class III: SIRT1-7; class IV: HDAC11) [19–21]. *In vivo* mouse studies utilizing HDAC inhibitors suggested that HDACs regulate the biological behaviors of conventional T cells and Foxp3^+^ Treg cells [22]. Moreover, previous data demonstrate that individual HDAC members also manage the development and function of specific T cell lineages. Among them, HDAC1 suppresses Th2 cytokine production in airway inflammation [23]. HDAC3 is required for the development of both iNKT cells and CD8^+^ memory T cells [24]. The nuclear export of HDAC7, which is indispensable for the negative and positive selection of the thymocytes, influences the expressions of adhesion molecules and cytokines along with their receptors involved in the function of cytotoxic T lymphocytes (CTL) [25] [26]. HDAC6, HDAC9 and Sirt1 are capable of mediating the histone deacetylation of the Foxp3 gene, thereby directing Treg cell functions [27, 28].

HDAC4, one member of the tissue-specific Class II HDACs, is highly expressed in neurons [29] and bone mass, and plays an essential role in maintaining neuronal survival [30] and chondrocyte hypertrophy [31]. Besides, nuclear HDAC4 distribution was enhanced in Purkinje neurons from Atm-deficient mice after lipopolysaccharides (LPS) stimulation, and Atm was identified to be involved in ataxia-telangiectasia characterized by immune deficiency [32], indicating that HDAC4 may directly or indirectly regulate inflammation genes. Ca2^+^-inducing release of the transcription factor MEF2, which plays an important role in T cell apoptosis [33], was regulated by HDAC4 [34]. However, the expression profile and function of HDAC4 in T cells are barely known.

In the current study, we discovered for the first time that HDAC4 is expressed in the multiple T cell lineages within the thymus. Using T-cell-specific HDAC4-ablated mice, we investigated the potential function of HDAC4 in the development and function of conventional T cells and iNKT cells.

## RESULTS

### HDAC4 is expressed in multiple T cell lineages

**Figure 1:**
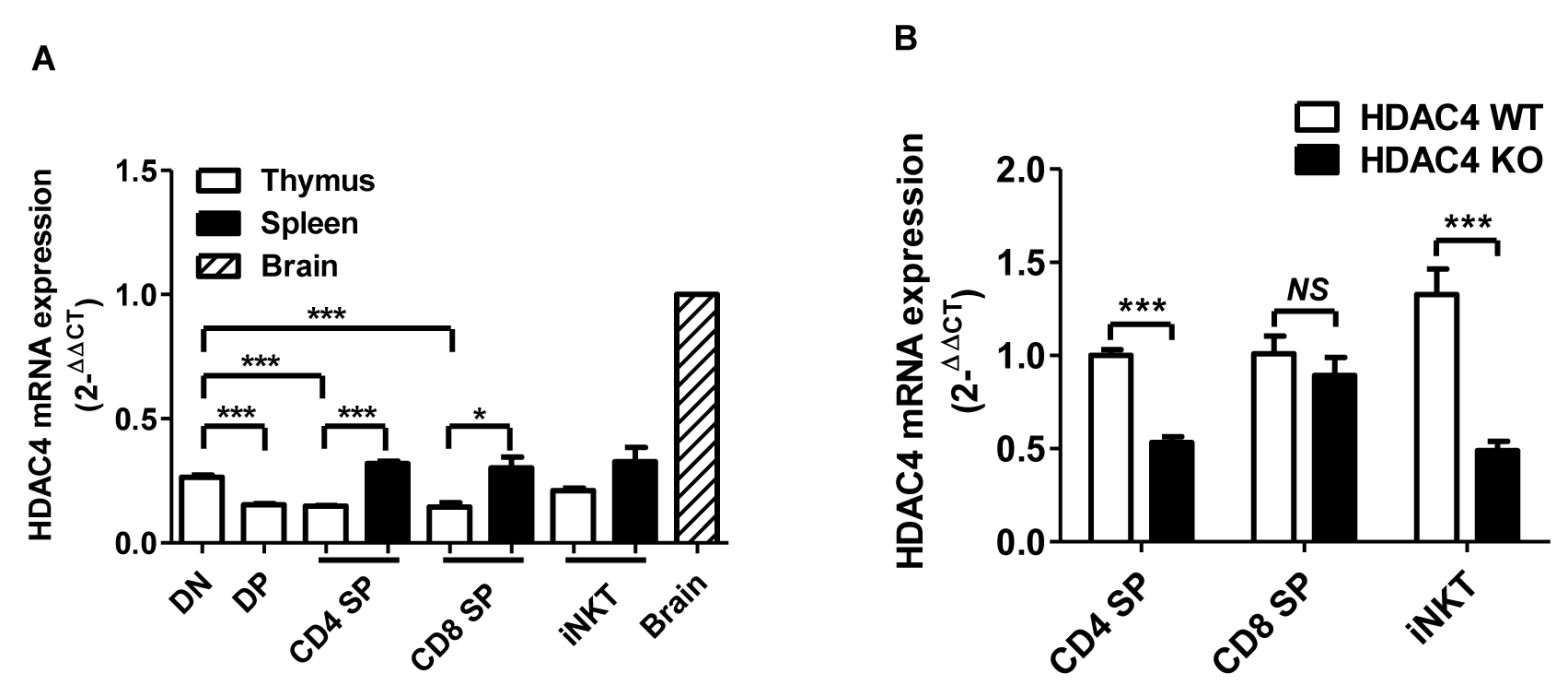
HDAC4 is expressed in multiple T cell lineages Thymic and splenic CD4^-^CD8^-^DN, CD4^+^CD8^+^DP, CD4^+^SP, CD8^+^SP, TCR-β^+^Tet^+^ iNKT cells from HDAC4 WT and HDAC4 KO mice were FACS sorted and examined for HDAC4 expression. **A**. Real-time PCR analysis of HDAC4 mRNA expression in sorted T cell and brain tissues. All samples were normalized to the HDAC4 expression in brain tissues. **B**. Real-time PCR analysis of HDAC4 deletion efficiency in sorted T cells from HDAC4 KO compared to WT. Data represents three independent experiments (mean ± SD). * *P* < 0.05, ** *P* < 0.01 and *** *P* < 0.001 (unpaired *t* test).

To detect HDAC4 expression in T cell lineages, thymus and spleen cells of wild-type (WT) mice were stained with CD4, CD8, TCR-β and CD1d-loaded tetramer (Tet). Different stages of T cells, based on their expressions of CD4 and CD8, and iNKT cells were sorted and assessed for HDAC4 mRNA expression by RT-PCR. As expected, HDAC4 was highly expressed in the brain tissue (Figure 1A). We discovered that HDAC4 was also expressed in multiple T cell subsets, including thymic CD4^-^ CD8^-^ DN and CD4^+^ CD8^+^ DP, thymic and splenic CD4^+^ SP cells and CD8^+^ SP T cells, as well as TCR-β^+^ Tet^+^ iNKT cells (Figure 1A). DN thymocytes expressed a higher level of HDAC4 compared to thymic DP, CD4^+^ SP and CD8^+^ SP T cells. Additionally, CD4^+^ SP and CD8^+^ SP T cells enhanced their expression of HDAC4 after migration to the spleen, whereas thymic and splenic iNKT cells displayed no significant difference in HDAC4 expression. Thus, HDAC4 is differentially expressed in conventional T cells and iNKT cells. And, the dynamic change of HDAC4 during T cell differentiation suggests its potential role in T cell development and function.

### Conventional T cells develop normally in the absence of HDAC4

**Figure 2:**
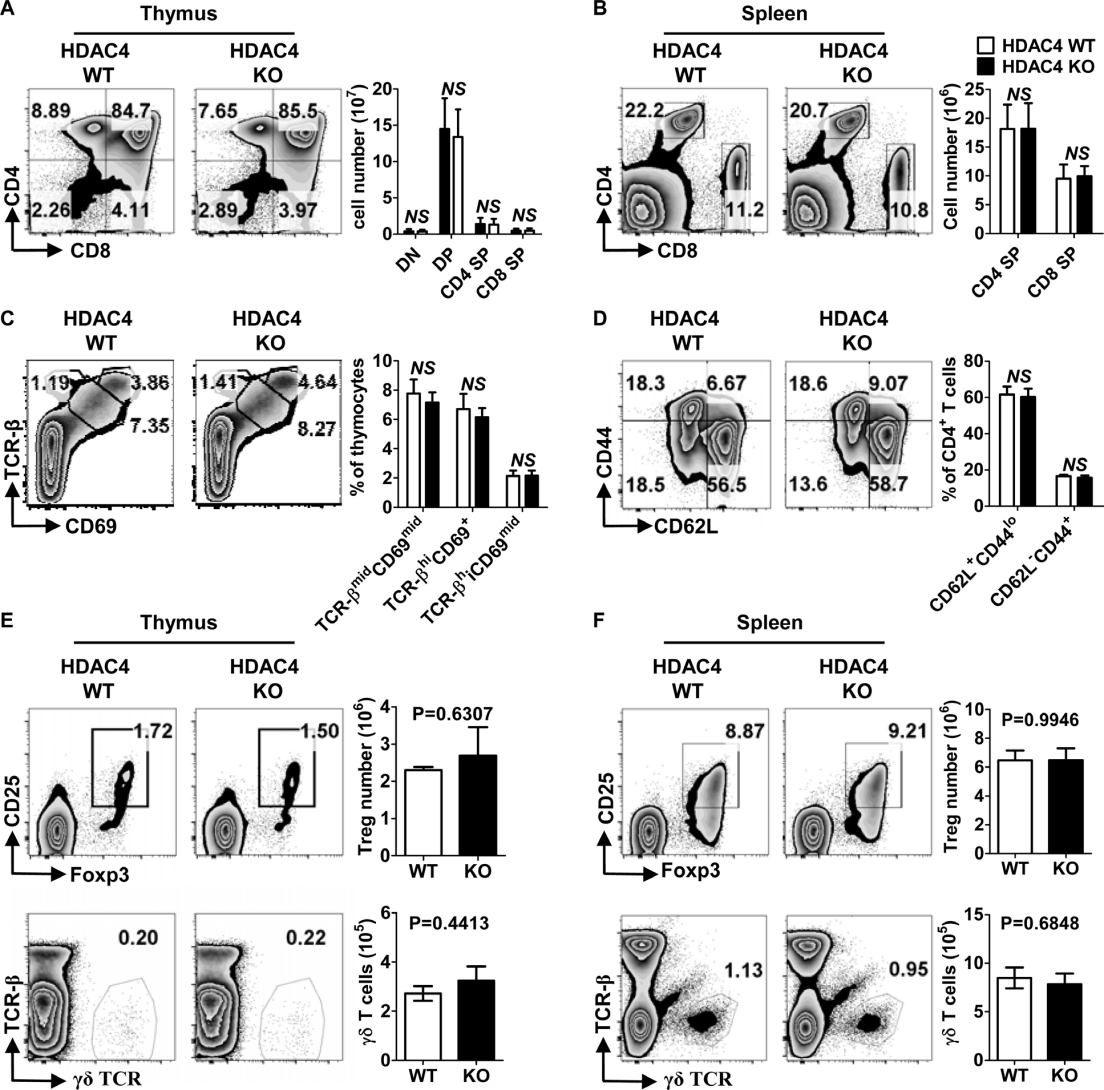
Conventional T cells developed normally in the absence of HDAC4 **A**., **B**. Flow cytometry analysis (left panel) and absolute numbers (right panel) of DN, DP, CD4 SP and CD8 SP based on CD4 and CD8 expression in thymus **A**. and spleen **B**. from HDAC4 WT and HDAC4 KO mice. **C**. Flow cytometry analysis (left panel) and frequencies (right panel) of T cell positive selection stages evaluated by TCR-β and CD69 expression on thymocytes from HDAC4 WT and HDAC4 KO mice. **D**. Flow cytometry analysis (left panel) and frequencies (right panel) of splenic CD62L^+^CD44^lo^ naïve and CD62L^-^CD44^+^ effector/memory CD4^+^ T cells from HDAC4 WT and HDAC4 KO mice are shown. **E**., **F**. Flow cytometry analysis (left panel) and absolute numbers (right panel) of CD25^+^Foxp3^+^ Treg cells of gated CD4^+^ T cells and γδ T cells in thymus **E**. and spleen **F**. are shown. Data represents three independent experiments with 2 to 3 mice per experiment (mean ± SD).

To assess the role of HDAC4 in T cell development, we generated T-cell-specific HDAC4-knockout (KO) mice by crossing loxp-flanked HDAC4 gene mutation HDAC4^fl/fl^ mice [35] with CD4-Cre transgenic mice. Mice that were homozygous for HDAC4^fl/fl^ with CD4-Cre expression were conditional HDAC4 ablation mice and designated as CD4^Cre^ HDAC4^fl/fl^ (HDAC4 KO), while the WT littermates were designated as HDAC4^fl/fl^ (HDAC4 WT). As shown in Figure 1B, HDAC4 expression was significantly reduced in thymic CD4^+^ SP T cells and iNKT cells from HDAC4 KO mice. Intriguingly, there was no significant decline in thymic CD8^+^ SP T cell expression of HDAC4. No significant alterations in the frequencies and absolute numbers of thymic CD4^-^CD8^-^ DN, CD4^+^CD8^+^ DP, CD4^+^ SP or CD8^+^ SP T cells were observed in HDAC4-deficient mice (Figure 2A). Similarly, the peripheral CD4^+^ and CD8^+^ T cell distribution was comparable between HDAC4 WT and KO mice (Figure 2B). Thymocytes that undergo positive selection are categorized into preselected (TCR-β^mid^ CD69^mid^), postselected (TCR-β^hi^ CD69^+^) and mature (CD69^mid^ TCR-β^hi^) thymocytes [36]. Equivalent percentages of different-stage thymocytes based on positive selection were identified between HDAC4 WT and KO mice (Figure 2C). Similar results were obtained when analyzing naïve (CD44^lo^ CD62L^+^) and effector/memory (CD44^hi^ CD62L^-^) CD4^+^ T cell subsets (Figure 2D). The frequency and absolute number of CD4^+^CD25^+^Foxp3^+^ Treg cells and γδ T cells residing in the thymus (Figure 2E) and spleen (Figure 2F) were also not affected in HDAC4 KO mice. Overall, loss of HDAC4 does not alter conventional T cell development.

### Normal iNKT cell development and maturation in HDAC4-deficient mice

**Figure 3:**
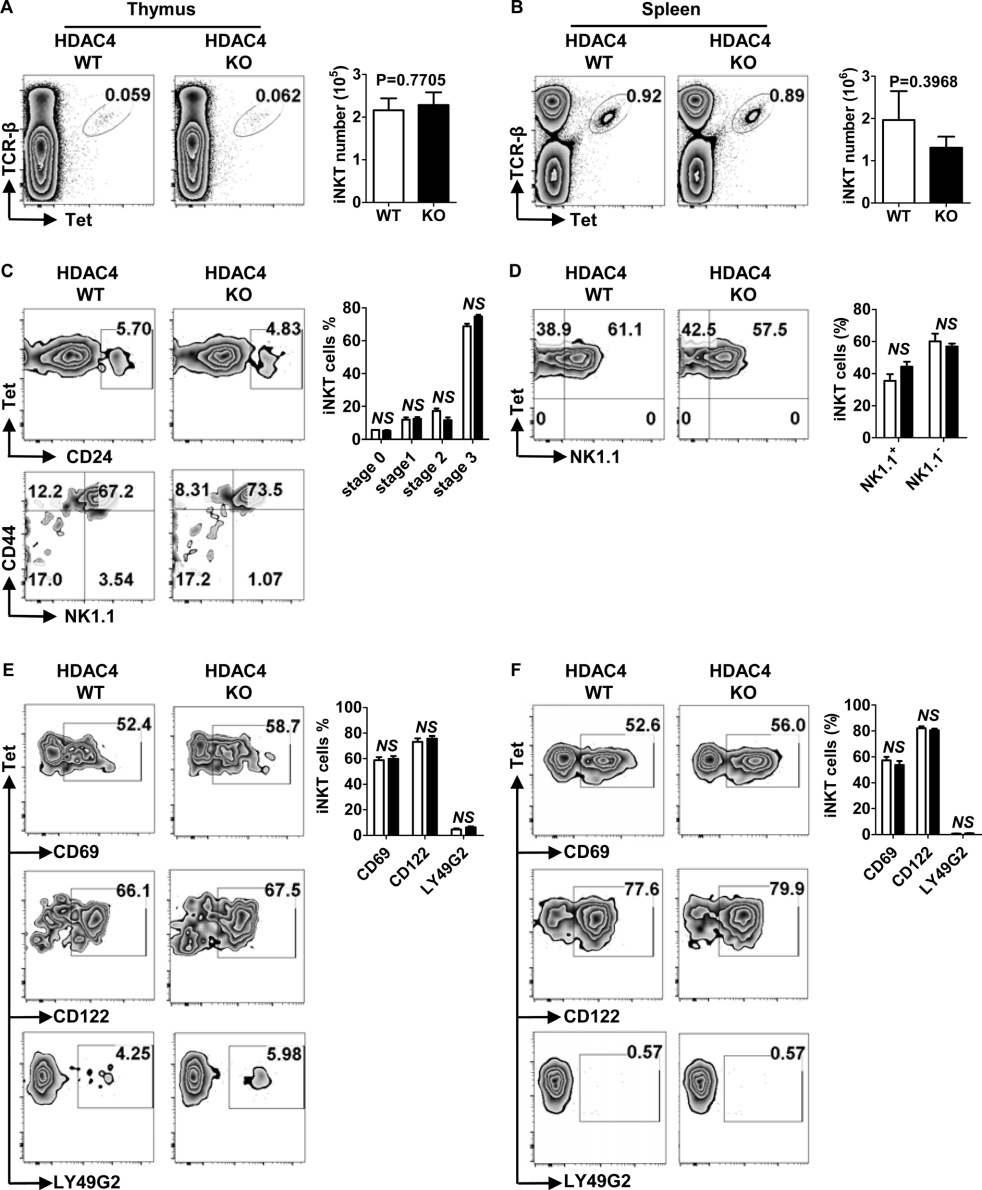
Normal iNKT cell development and maturation in HDAC4-deficient mice **A**., **B**. Flow cytometry analysis (left panel) and absolute numbers (right panel) of TCR-β^+^Tet^+^ iNKT cells in thymus **A**. and spleen **B**. from HDAC4 WT and HDAC4 KO mice are shown. **C**., **D**. Flow cytometry analysis (left panel) and frequencies (right panel) of iNKT cell maturation stages based on CD24, CD44, NK1.1 expression of thymic iNKT cells **C**. and NK1.1 expression of splenic iNKT cells **D**. are shown. **E**., **F**. Flow cytometry analysis (left panel) and frequencies (right panel) of gated iNKT cells expressing CD69, CD122 or LY49G2 in thymus **E**. and spleen **F**. from HDAC4 WT and HDAC4 KO mice are shown. Data represents three independent experiments with 2 to 3 mice per experiment (mean ± SD).

In addition to conventional T cells, thymocytes can also differentiate into CD1d-dependent iNKT cells, which mediate immune homeostasis and maintain self-tolerance [9, 10]. We discovered no significant differences in the percentages and absolute numbers of iNKT within the thymus (Figure 3A) and spleen (Figure 3B) between HDAC4 KO mice and their WT counterparts. We further investigated if HDAC4 was required for iNKT cell maturation. As shown in Figure 3C, thymic iNKT cell frequencies of distinct developmental stages were all comparable between HDAC4 WT and KO mice. Upon iNKT cell maturation, the expression of certain surface receptors are also upregulated, including CD69, LY49G2 and CD122. As shown in Figure 3E, the positive rates of these maturation markers expressed on iNKT cells were not significantly different between HDAC4 WT and KO mice. During iNKT cell maturation, most thymic immature NK1.1^-^ iNKT cells emigrate to peripheral tissues where they rapidly mature into NK1.1^+^ iNKT cells. To further investigate whether peripheral iNKT cell maturation was defective in HDAC4-deficient mice, we analyzed splenic iNKT cell maturation status by assessing the expression levels of maturation markers. Consistent with thymus results, splenic iNKT cells were fully mature in HDAC4-deficient mice (Figure 3D and 3F). Taken together, HDAC4 is not required for iNKT cell development and maturation.

### Unaltered Th1, Th2 and Th17 cell differentiation in HDAC4-deficient mice

**Figure 4:**
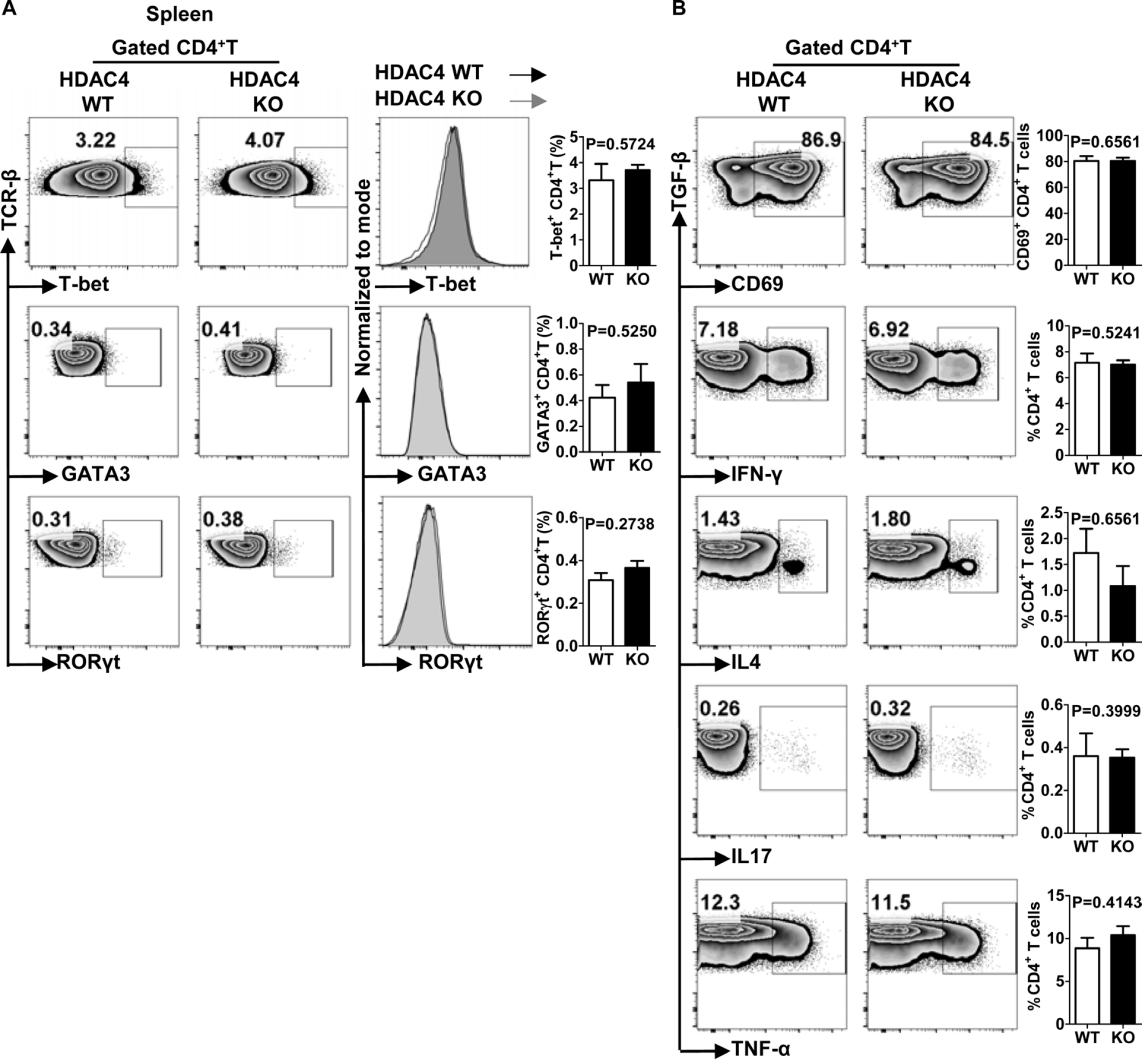
Unaltered Th1, Th2 and Th17 cell differentiation in HDAC4-deficient mice **A**. Flow cytometry analysis (left panel), histogram (middle panel) and frequencies (right panel) of T-bet, GATA3 and RORγt expressions in splenic conventional CD4^+^ T cells from HDAC4 WT and HDAC4 KO mice are shown. **B**. Whole splenocytes from HDAC4 WT and HDAC4 KO mice were treated with PMA and ionomycin *in vitro* for 4 h. Flow cytometry analysis (left panel) and frequencies (right panel) of CD69, IL4, IFN-γ, IL17 and TNF-α expression of CD4^+^T cells from HDAC4 WT and HDAC4 KO mice are shown. Data represents three independent experiments with 2 to 3 mice per experiment (mean ± SD).

To investigate whether HDAC4 regulated the polarization of T helper (Th) cells, we examined the expression levels of house-keeping transcription factors for each Th cell lineage. As shown in Figure 4A and 4B, the frequencies of T-bet-, GATA-3- and RORγt-positive iNKT cells along with their mean fluorescence intensities (MFI) were equivalent between HDAC4 WT and KO mice. Previous studies reveal an enhancement of histone acetylation at the IFN-γ and IL-4 gene loci in respective Th1 and Th2 cell lineages, implying that HDACs might regulate the cytokine-secreting property of Th cells [17, 37]. To further explore the role of HDAC4 in Th cell function, the splenocytes of HDAC4 WT and KO mice were stimulated *in vitro* with phorbol 12-myristate 13-acetate (PMA) and ionomycin for 4 hours. In accordance with the unaffected expression of transcription factors, there was no significant difference in the production of TNF-α, IFN-γ, IL-4 or IL-17 by Th cells nor in their CD69 expression between HDAC4 WT and KO mice (Figure 4C). Thus, HDAC4 is not essential in conventional T cell polarization and function.

### HDAC4 deficiency does not affect iNKT cell polarization

**Figure 5:**
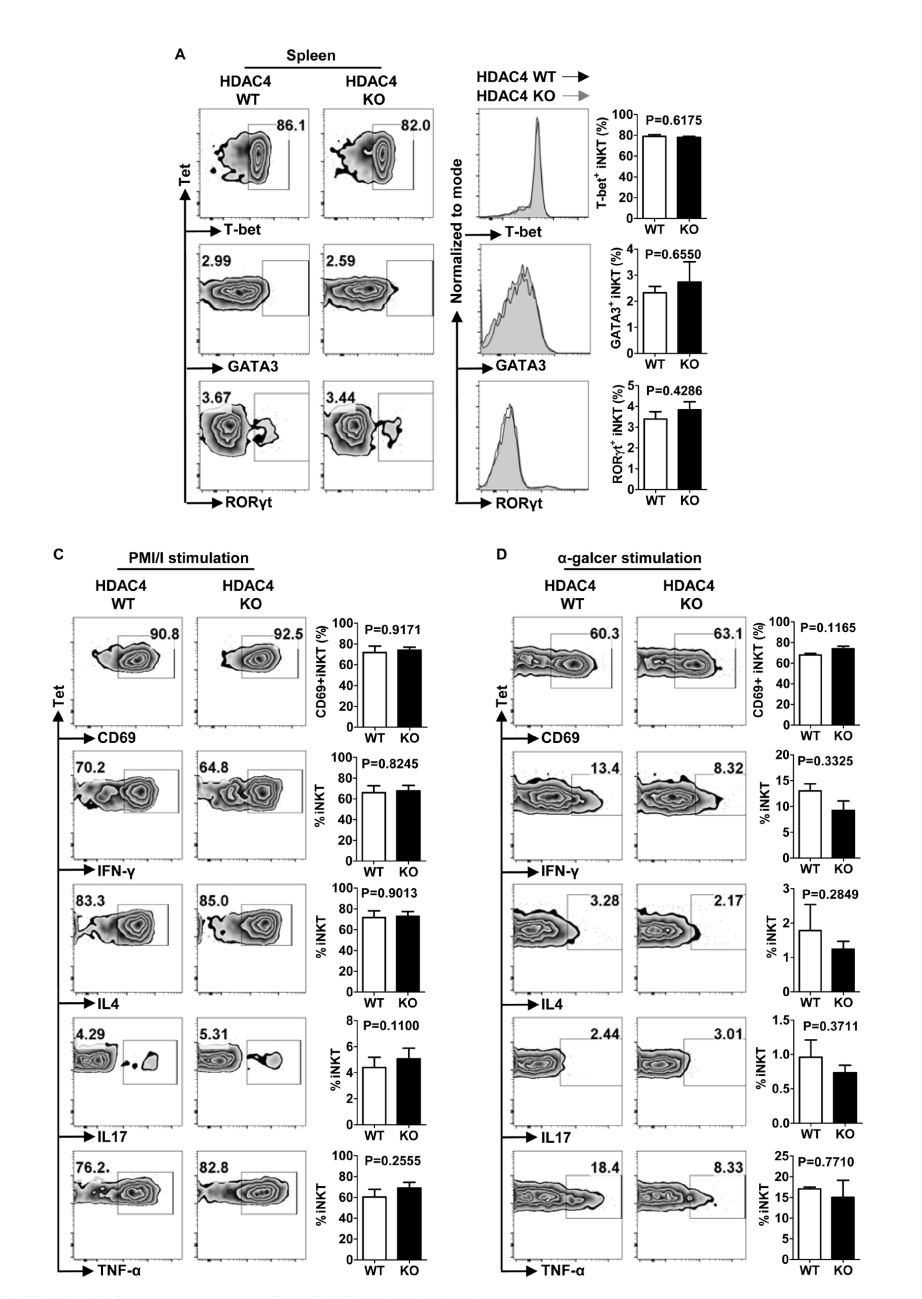
HDAC4 deficiency does not affect iNKT cell polarization **A**. Flow cytometry analysis (left panel), histogram (middle panel) and frequencies (right panel) of T-bet, GATA3 and RORγt expressions in splenic iNKT cells from HDAC4 WT and HDAC4 KO mice are shown. **C**., **D**. Flow cytometry analysis (left panel) and frequencies (right panel) of CD69, IL4, IFN-γ, IL17 and TNF-α expression of splenocytes with *in vitro* 4 hours PMA and ionomycin stimulation **C**. or *in vivo* 2 hours α-Galcer stimulation **D**. from HDAC4 WT and HDAC4 KO mice are shown. Data represents three independent experiments with 2 to 3 mice per experiment (mean ± SD).

iNKT cell subsets, including NKT1, NKT2 and NKT17, share analogous transcription factor expression and cytokine production with the correspondent conventional Th cell sub-lineages. Flow cytometry analysis uncovered no significant change in T-bet, GATA-3 or RORγt expression by thymus and spleen iNKT cells upon HDAC4 ablation (Figure 5A, 5B). Further investigation with *in vitro* PMA/Ionomycin stimulation showed competent cytokine-producing ability in HDAC4-deficient iNKT cells (Figure 5C). To specifically stimulate iNKT cells *in vivo*, we injected mice with α-GalCer. After 2.5 hours of stimulation, the secretion of IFN-γ, IL4, IL17 and TNF-α as well as the CD69 expression in splenic iNKT cells were also comparable between HDAC4 WT and KO mice (Figure 5D). Overall, these data suggest that loss of HDAC4 does not disturb the polarization of iNKT cell subsets.

## DISCUSSION

Class IIa HDACs are characterized by their tissue-specific expression manner, which have been reported to exert transcriptional repressive function in bone, heart, smooth muscle and brain where they mediate tissue development. HDAC4, a member of the Class IIa HDACs, has been identified to be highly expressed in neurons [29] and to profoundly influence neuronal development and function [38, 39]. A series of studies has established a potential link between immune regulation and HDAC4. The expressions of multiple immune-related transcription factors, including c-Jun [40], NF-κB, and Bcl-6 [41, 42], are controlled by HDAC4. HDAC4 is also required for the inhibitory effect of nuclear liver X receptor (LXR)-mediated repression of the inflammatory response in LPS-activated mouse macrophages [43]. Moreover, macrophage-specific HDAC4 deletion enhances adipose-tissue macrophage infiltration in response to high fat diet [44]. In a mouse model of emphysema, HDAC4 was targeted by miR-22 to promote a Th17 response in antigen presenting cells (APCs) [45].

However, the role of HDAC4 in T cell lineages remained unclear. To the best of our knowledge, we are the first to report that HDAC4 is differentially expressed in conventional T cells and iNKT cells residing in various lymphoid organs. A previous research study uncovered that nuclear export of HDAC4 was essential in provoking IL-5 expression in activated human T Jurkat cells [46]. However, we reported here that HDAC4 ablation in T cells did not affect conventional T cell development, maturation or cytokine-secreting function. Nevertheless, the role of HDAC4 in other T cell lineages including TFH (T follicular helper) cell [47] and Th9 [48] cell were unclear, which need further investigation in the future. In addition, HDAC4 is associated with human T cell relevant disorders. In T cell acute lymphoblastic leukemia (ALL) patients, hyperexpression of HDAC4 is correlated with a higher initial count of leukocytes and poor response to prednisone [49]. And, HDAC4 is one of the most hyper-methylated genes outside the MHC region on CD4^+^ T cells from rheumatoid arthritis patients [50]. Our study focused on the steady-state role of HDAC4 in T cell immunity, but the function of HDAC4 in T cell reaction under inflammation conditions is still undetermined, and thus needs further investigation.

Previous data revealed that the inhibition of class II HDACs alone (MC1568) or together with class I HDACs (TSA and LBH589) can enhance tumor cell CD1d expression and their antigen-presenting capability [51, 52]. Among the Class I HDAC members, HDAC1 and HDAC2 can modulate CD1d-dependent NKT cell hybridoma response [51]. The loss of HDAC2 in NKT-like cells is associated with lymphocyte senescence in chronic obstructive pulmonary disease [53]. In addition, T-cell-specific deletion of HDAC3 leads to a block of iNKT cell development [54]. However, the role of individual Class II HDAC members in iNKT cell biology was unclear. Utilizing T-cell-specific HDAC4-deficient mice, our data implied that lack of HDAC4 does not affect iNKT cell development, maturation, or cytokine production. Nevertheless, other unexamined properties of iNKT cells, including anergy induction, uncommon sublineage polarization [55] and disease-associated immune functions [56] need further exploration.

Although Class IIa HDACs possess a highly-conserved catalytic domain, they exhibit minimal deacetylase activity on acetylated histones which demand recruiting other proteins (corepressors or coactivators) to modulate gene expression. In fact, the enzymatic activity of HDAC4 depends on the formation of a multiprotein complex containing HDAC3 and silencing mediator for retinoid and thyroid receptors/nuclear receptor corepressor (SMRT/N-CoR) [57]. The dependence of HDAC4 on other transcription regulators suggest a redundant role of HDAC4 with other HDACs in mediating T cell lineages, especially HDAC3, which is probably the major regulator among HDAC family members in T cell maturation and iNKT cell development [24, 54, 58].

In conclusion, the analysis of HDAC4 conditional knockout mice indicates that HDAC4 deficiency does not alter conventional T cell and iNKT cell development, maturation, polarization and function, indicating that HDAC4 is not a key gene in regulating conventional T cell and iNKT cell biology.

## MATERIALS AND METHODS

### Mice

The floxed HDAC4 was described previously [35]. C57BL/6 and CD4-Cre mice were purchased from The Jackson Laboratory. In this study, all wildtype (WT) control mice CD4Cre-HDAC4^fl/fl^ (defined as HDAC4 WT) were littermates of CD4Cre+HDAC4^fl/fl^ mice (defined as HDAC4 KO). 6 to 8 weeks old age and sex-matched mice were used. Handling of mice and experimental procedures were in accordance with requirements of the Institutional Animal Care and Use Committee.

### Genotyping

CD4Cre HDAC4^fl/fl^ mice were genotyped using the following PCR primer pairs: CD4 Cre: 5’-GCATTTC TGGGGATTGCTTA-3’ and 5’-GTCATCCTTAGCGC CGTAAA’. HDAC4^fl/fl^: 5’-ATCTGCCCACCAG AGTATGTG-3 ‘, 5’-CTTGTTGAGAACAAACTCC TGCAGCT-3’, 5’ - GATTGACCGTAATGGG ATAGGTTACG-3’.

### Flow cytometry and antibodies

Single-cell suspensions were incubated with anti-FcγRII/III (clone 2.4G2) for 10 minutes at 4 °C. Cells were stained for surface and intracellular markers with conjugated monoclonal antibodies listed as below: CD4 (RM4-5), CD8 (53-6.7), TCR-β (H57-597), NK1.1 (PK136), CD24 (M1/69), CD44 (IM7), CD62L (MEL-14), CD122 (5H4), CD69 (H1.2F3), LY49G2 (LGL-1), B220 (RA3-6B2), Foxp3 (FJK-16s), CD25 (PC61), T-bet (ebio4B10), RORγt (B2D), GATA3 (TWAJ). All these mAbs were purchased from eBioscience (San Diego, CA, USA) or Biolegend (San Diego, CA, USA). Lipid PBS-57 (an analogue of α-GalCer)-loaded murine CD1d tetramers were provided by the National Institutes of Health Tetramer Facility (Atlanta, GA, USA). Data were analyzed using FlowJo 10.0.0 software.

### Cell stimulation *in vitro* and intracellular cytokine assays

Total splenocytes were cultured in RPMI 1640 (Life Technologies) supplemented with 10% FCS and stimulated with PMA (50ng/ml, LC laboratory) plus Ionomycin (747ng/ml, LC laboratory). 1.5 hours later cells were cultured for an additional 2.5 h in the presence of Protein Transport Inhibitor Brefeldin A (1μl/ml, eBioscience). After incubation, cells were harvested and washed once with 1× PBS. After the surface marker staining, cells were fixed and permeabilized with IC Fixation Buffer (eBioscience) and then stained for intracellular anti-IL-4 (11B11), IL17 (TC11-18H10.1), TNF-α (MP6-XT22) and IFN-γ (XMG1.2).

### α-GalCer-induced cell stimulation *in vivo*

Splenocytes were collected at 2.5 hours after α-galcer (2μg/mouse) injection (intravenous) and cultured in the presence of Brefeldin A (1μl/ml, eBioscience) for an additional hour. After incubation, cells were harvested and stained for surface markers and intracellular cytokines by the procedures mentioned above.

### DN, DP, CD4^+^SP, CD8^+^SP and iNKT cell sorting

Single cell suspensions of thymocytes and splenocytes were obtained from 8 week old HDAC4 WT and HDAC4 KO mice. DN, DP, CD4^+^SP, CD8^+^SP T cells and NKT cells were sorted after anti-CD4, anti-CD8, anti-TCR-β and CD1d-tetramer antibody staining by BD FACSArialII (BD Biosciences). Sorted cells were collected with at least 95% purity.

### Real-time RT-PCR for mRNA expression

Total RNA from DN, DP, CD4^+^SP, CD8^+^SP T cells, iNKT cells and brain tissue were harvested using the Qiagen mRNA isolation kit (Qiagen) according the manufacturer's instructions. Realtime PCR was performed in triplicates using FastStart Universal SYBR Green Master (Roche Diagnostics Corp., Indiana, USA) on ABI 7900 HT Real-Time PCR system (Applied Biosystems, Foster City, CA, USA). Data were normalized to mouse GAPDH mRNA.

### Statistical analysis

Statistical analysis was performed with Prism 5.0 (GraphPad Software). The two-tailed Student t test was used. Differences were considered statistically significant when values of *p* < 0.05.
